# Exercise as a geroprotector: focusing on epigenetic aging

**DOI:** 10.18632/aging.206278

**Published:** 2025-07-08

**Authors:** Takuji Kawamura, Mitsuru Higuchi, Zsolt Radak, Yasuyuki Taki

**Affiliations:** 1 Smart Aging Research Center, Tohoku University, Miyagi 980-8575, Japan; 2 Faculty of Sport Sciences, Waseda University, Saitama 359-1192, Japan; 3 Research Institute of Molecular Exercise Science, Hungarian University of Sports Science, Budapest H-1123, Hungary; 4 Faculty of Sciences, Institute of Sport Sciences and Physical Education, University of Pécs, Pécs, Hungary; 5 Department of Bioengineering, Sapientia Hungarian University of Transylvania, Miercurea Ciuc 530104, Romania

**Keywords:** physical activity, exercise, physical fitness, epigenetic clock, geroprotector

## Abstract

Emerging evidence suggests that physical activity, exercise, and physical fitness may delay or reverse epigenetic aging, with implications for the extension of healthspan. This Perspective review defines essential exercise-related terminology and synthesizes findings from both human and animal studies examining the relationships between these factors and DNA methylation-based epigenetic clocks. While observational studies have demonstrated inverse relationships between cardiorespiratory fitness and epigenetic age acceleration, interventional studies further suggest that structured exercise training can induce epigenomic rejuvenation, particularly in blood and skeletal muscle. However, these effects exhibit considerable interindividual and organ-specific variability, underscoring the need for future research to elucidate causal mechanisms and organ-specific responses in order to optimize the application of exercise as a geroprotective intervention.

## INTRODUCTION

Aging is an unavoidable biological process characterized by a gradual decline in physiological function and an increased susceptibility to disease. Recent advances in aging research have focused on the role of epigenetic mechanisms, particularly DNA methylation patterns, in the regulation of aging. The concept of an epigenetic clock is a predictive model based on DNA methylation patterns that provides a more accurate estimate of biological age than chronological age [1, 2]. Physical activity has emerged as a modifiable lifestyle factor that can influence the epigenetic clock and may serve as a geroprotective intervention to extend the health span and possibly the life span. However, some studies have discussed these effects without clearly distinguishing between physical activity, physical fitness, and exercise, which are closely related terms [3]. In this Research Perspective, we clarify the definitions of these terms and outline the literature on their relationship with epigenetic aging. We also reviewed the findings of both human and animal studies and discussed the systemic effects of exercise on the epigenetic clock. In addition, we identify future directions and challenges in this field.

### Fundamental terms of exercise science

Understanding the effects of exercise on aging requires an understanding of the basic terms used in exercise science (Table 1). Physical activity refers to any bodily movement produced by skeletal muscles that results in energy expenditure. Conversely, exercise is a subset of physical activity that is planned, structured, and repetitive, with the final or intermediate goal of improving or maintaining physical fitness [3]. Physical fitness is also a term used to describe a set of attributes that people have or achieve that relates to their ability to perform physical activities [3]. These foundational terms–physical activity, exercise, and physical fitness–are often used interchangeably in the general population; however, they have distinct physiological and epidemiological implications, particularly in aging research. For instance, while light-intensity physical activity, such as casual walking, contributes to energy expenditure and general health maintenance, it may not provide a sufficient stimulus to induce the molecular and cellular adaptations typically associated with geroprotective effects. In contrast, structured exercise programs, especially those incorporating moderate-to-vigorous intensity, are more likely to elicit systemic responses such as improved mitochondrial function, enhanced insulin sensitivity, and modulation of epigenetic markers. Furthermore, physical fitness, particularly cardiorespiratory fitness (CRF) and muscular strength, has been shown to be a robust predictor of morbidity and mortality in older adults [7–10]. It is important to note that while physical activity and exercise are behaviors, physical fitness represents an integrated outcome influenced by genetics, training status, and overall health. Therefore, when evaluating the impact of exercise on epigenetic aging, distinguishing between these terms allows for a more precise interpretation of study findings and the development of targeted interventions.

**Table 1:** The definitions: physical activity, exercise, and physical fitness [3–6].

**Term**	**Definition**	**Characteristics**	**Examples**
Physical activity	Any bodily movement produced by skeletal muscles that results in energy expenditure.	Broad concept; includes all movements in daily life.	Walking, climbing stairs, cleaning, commuting
Exercise	A subcategory of physical activity that is planned, structured, repetitive, and purposeful, typically aimed at improving or maintaining physical fitness.	Subset of physical activity; intentional and goal-directed.	Jogging, weight training, swimming, yoga
Physical fitness	A set of health- and skill-related attributes related to the ability to perform physical activity, influenced by both genetic factors and training.	Represents a physiological outcome rather than a behavior.	VO2 max, muscular strength, flexibility, endurance

Abbreviation: VO_2_ max: maximal oxygen uptake.

### The relationship between physical activity and epigenetic aging

Several studies have investigated the relationship between physical activity and epigenetic aging, in contrast to the fewer investigations into the roles of the other two concepts, physical fitness and exercise. At the forefront of this field, Quach et al. reported a weak negative association between self-reported physical activity and epigenetic age acceleration [11]. A subsequent study by Gale et al. reported that sedentary and walking behaviors, objectively measured using accelerometers, were not associated with epigenetic age acceleration [12]. In a study by Sillanpaa et al., which included twin pairs with leisure-time physical activity (LTPA) discordance, there was no difference in epigenetic age acceleration between active and inactive twins [13]. Although previous studies have shown no or weak associations between physical activity and epigenetic aging, numerous subsequent studies have reported negative associations [14–20]. Both self-reported [14, 15, 19, 20] and accelerometer-based assessments [16, 17, 18] of physical activity have shown a negative association with epigenetic aging. In contrast, sedentary behavior has been suggested to accelerate epigenetic aging [18, 20]. It has also been shown that LTPA and occupational physical activity (OPA) seem to have different effects, that is, LTPA is negatively associated with epigenetic aging and is positively associated with epigenetic aging [14]. Collectively, these findings suggest that increased leisure-time physical activity and reduced sedentary behavior may have beneficial effects on epigenetic aging.

### The effects of exercise training on epigenetic aging: evidence from animal and human studies

Long-term exercise training interventions are essential for establishing a causal relationship between exercise and epigenetic aging. In animal studies, Murach et al. subjected 22- to 24-month-old mice to eight weeks of voluntary endurance/resistance exercise training (progressive weighted wheel running, PoWeR) and found that age-related hypermethylation of promoter regions and epigenetic clock progression were slightly suppressed in the skeletal muscles of the exercise-trained group [21]. A study by the same group found that eight weeks of late-life exercise training (PoWeR) was associated with lower skeletal muscle mDNAge using a contemporary muscle-specific epigenetic clock [22]. In human studies, Voisin et al. showed that exercise training helps retain a more youthful methylome and gene expression profile in skeletal muscles. [23]. In the study by da Silva Rodrigues et al., sedentary middle-aged and older females underwent eight weeks of combined (aerobic and strength) training. The group with a higher epigenetic age prior to the intervention showed a significant decrease in epigenetic age after the intervention [24]. These findings suggest that structured exercise training can effectively reverse or rejuvenate blood- and skeletal muscle-based epigenetic clock(s) and the aging methylome.

### The relationship between physical fitness and epigenetic aging

Few studies have examined the relationship between physical fitness obtained through exercise and epigenetic aging. McGreevy et al. developed DNAmFitAge, which incorporates physical fitness measures into DNA methylation data, and found that bodybuilders had significantly lower DNAmFitAge compared to age-matched controls [25]. Jokai et al. estimated maximal oxygen uptake values based on the Chester step test, assigned them to medium-to-low-fit and high-fit groups, and compared epigenetic aging [26]. The results showed that epigenetic age acceleration in the high-fitness group was 1.5 and 2.0 years lower in males and females, respectively, than in the medium-to-low-fitness group. A unique study comparing Olympic champions and non-champions who performed rigorous and long-term exercise from adolescence to adulthood found that epigenetic age acceleration was lower in Olympic champions than in non-champions [27]. Our study examined the relationship between physical fitness variables, such as peak oxygen uptake (VO_2_ peak), oxygen uptake at the ventilation threshold (VO_2_ at VT), which is the point of exercise intensity when breathing increases rapidly during exercise and the secretion of various endocrine hormones increases, grip strength, leg extension power, and epigenetic aging in older males [28]. We found a negative correlation between VO_2_ peak, VO_2_ at VT, and epigenetic age acceleration, even after adjusting for age, smoking, and alcohol consumption status. In addition, VO_2_ peak and VO_2_ at VT were more strongly associated with epigenetic age acceleration than grip strength and leg extension power. Furthermore, individuals whose fitness levels (i.e., VO_2_ peak and VO_2_ at VT) were above the reference values showed lower epigenetic age acceleration than those whose fitness levels were below the reference values. Cordero et al. investigated the association between blood DNA methylation profiles and CRF in 78 participants (including those with chronic airflow limitation: CAL) aged ≥ 40 years and found that higher CRF was associated with lower epigenetic age acceleration, and this effect was consistently observed in individuals with CAL [29]. These findings suggest that maintaining a high level of physical fitness delays epigenetic aging; however, these studies did not establish a causal relationship.

### Which organs are the targets of exercise-induced geroprotective effects?

Most human studies have measured DNA methylation in blood and skeletal muscle samples. As mentioned in a previous Editorial [30], it is well known that the beneficial effects of exercise are systemic [31, 32]. Therefore, it is of scientific interest to identify the target organs of the geroprotective effects of exercise. It is reasonable to assume that the primary target organ is the skeletal muscle; however, a recent report using a rat model selectively bred for high and low CRF revealed that the group with high CRF had a lower epigenetic age in the adipose tissue, cardiac muscle, and liver, in addition to the skeletal muscle, compared to the group with low CRF [33]. In our latest study, we used a similar rat model to compare the indicators of epigenetic aging in multiple organs of rats with high and low CRF. [34]. We observed that epigenetic clocks trained with available rat blood-derived data did not reflect differences in CRF in any of the organs, including the hippocampus, heart, skeletal muscle, or large intestine. In contrast, organ-specific differences owing to CRF were observed in the global mean DNA methylation in the soleus muscle and mean methylation entropy in the heart and large intestine, and the direction of these differences was opposite to that of age-related changes in rat blood. These findings suggest that maintaining physical fitness delays epigenetic aging in multiple organs and supports the notion that exercise as a geroprotector confers benefits to various organs. Moreover, recent findings indicate that the gut microbiome is associated with both epigenetic age acceleration and physical fitness [35], underscoring the need for continued research on inter-organ aging networks and the effects of exercise on these networks.

### Future challenges

**Figure 1:**
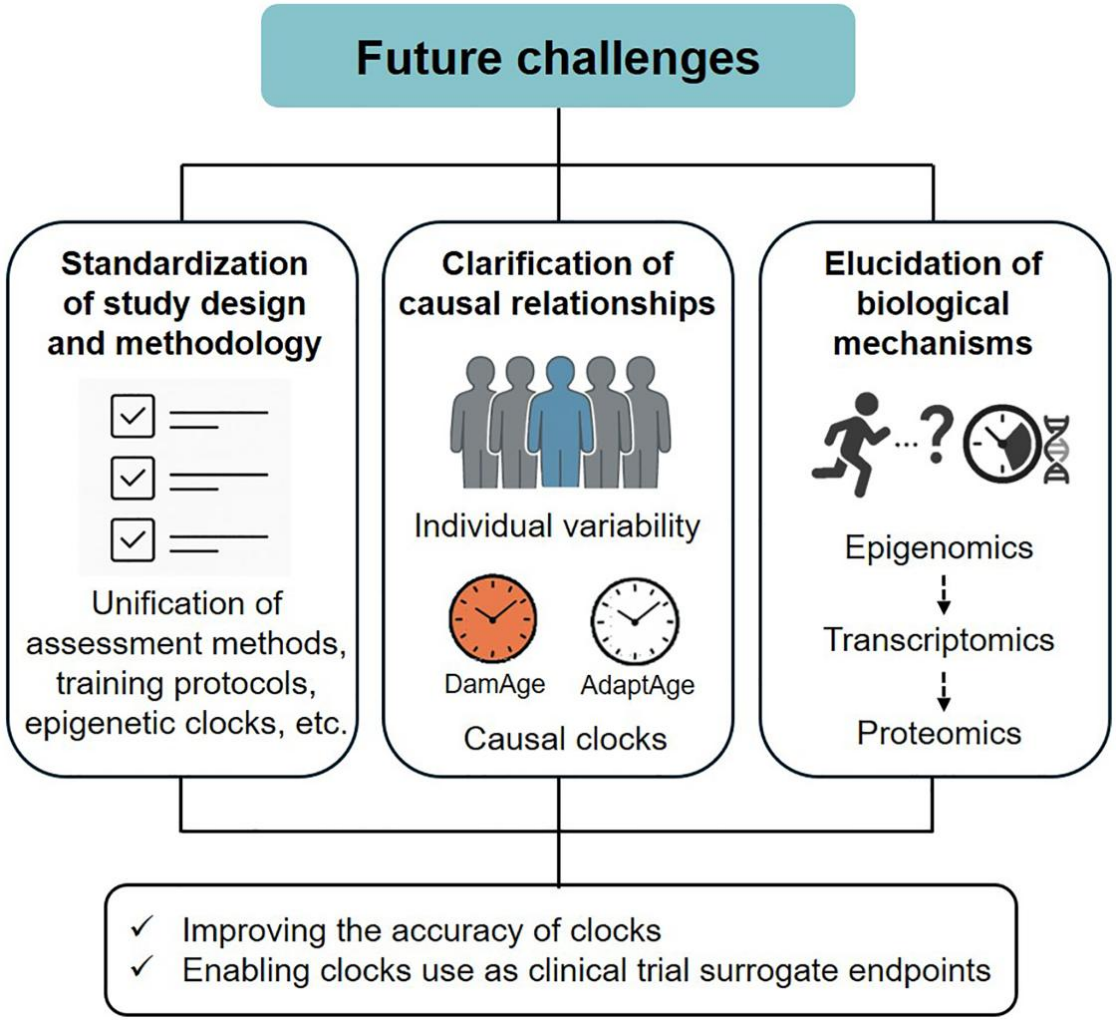
Future challenges overview.

As described above, physical activity, physical fitness, and exercise may be beneficial in delaying or reversing epigenetic aging in various organs. Considering the limited scientific evidence in this field, this section outlines the future research directions (Figure 1). The accuracy of the epigenetic clock and the issue of its use as a surrogate endpoint for clinical trials were excluded from the discussion, as these topics have been discussed elsewhere [36, 37]. First, the standardization of study design and methodology is essential to improve reproducibility and comparability across studies. This includes the unification of physical activity, physical fitness, and exercise assessment methods, training protocols, and epigenetic clock selection. It is also necessary to conduct studies with diverse subjects and populations (e.g., age, sex, and race). Second, further clarification of causal relationships based on individual variability is required. As noted above, responsiveness to exercise training may be greater in individuals with a higher epigenetic age at baseline [24]. Similarly, in a recent study, epigenetic age acceleration in response to exercise training did not change significantly in the overall average, but there was large individual variability, which also tended to improve, especially in those with higher pre-intervention age acceleration values (unpublished data). These findings suggest that the response of epigenetic aging to exercise is not homogeneous, and personalized exercise interventions are promising. More recently, causal epigenetic clocks (i.e., DamAge and AdaptAge) have been developed using CpG sites associated with age-related DNA methylation changes, which are presumed to have causal effects on health and diseases [38]. Therefore, testing whether exercise interventions alter the methylation status of these CpGs may reveal the causal basis for the geroprotective effects of exercise. Third, it is essential to understand the biological mechanisms by which exercise regulates epigenetic aging. The epigenetic clock, which is currently the dominant molecular biomarker for predicting biological age, was constructed without considering the molecular mechanisms underlying age-related DNA methylation changes [37]. Acute and long-term exercise are known to affect both genome-wide changes in CpG methylation (global methylation) and local methylation changes in specific gene promoter regions, primarily in the skeletal muscle [39]. However, the biological mechanisms linking exercise and epigenetic clocks are currently unknown, and future studies will require detailed observations of the effects of exercise on methylation levels at CpG sites that constitute the clock and gene expression.
